# Effect of management system and pedoclimatic factors on *Fusarium* and *Didymella* communities associated with pea (*Pisum sativum*) roots in Germany

**DOI:** 10.1038/s41598-025-86018-7

**Published:** 2025-01-21

**Authors:** Adnan Šišić, Jelena Baćanović-Šišić, Harald Schmidt, Maria R. Finckh

**Affiliations:** 1 Section of Ecological Plant Protection, University of Kassel, 37213 Witzenhausen, Germany; 2 Foundation Ecology & Agriculture (SOEL), 53474 Ahrweiler, Germany

**Keywords:** Germany, Pea root rot, Organic agriculture, Conventional agriculture, Spring peas, Winter peas, Pathogens, Microbe, Fungi, Fungal ecology, Fungal genetics

## Abstract

From 2016 to 2019, 128 organic and conventional spring and winter pea fields in Germany were surveyed to determine the effects of cropping history and pedo-climatic conditions on pea root health, the diversity of *Fusarium* and *Didymella* communities and their collective effect on pea yield. Roots generally appeared healthy or showed minor disease symptoms despite the frequent occurrence of 4 *Didymella* and 14 *Fusarium* species. Soil pH interacted with the occurrence of the *Fusarium oxysporum* species complex (FOSC) and *F. tricinctum* that correlated with reduced or increased soil pH values, respectively. While legumes in the cropping history or reduced time between legumes correlated with occurrence of *D. pinodella* and to a lesser degree with the members of the *F. solani* species complex (FSSC), the reverse was true at least in organic spring peas for *F. redolens*. Only in conventional systems increased root infections with *F. redolens* and the FSSC were linked to root rot incidence whereas yields negatively correlated with the FOSC and positively with *F. tricinctum* isolation frequencies. Overall, this study shows that pea root rot pathobiome is rather stable and that the damage caused is mostly due to the interaction with environmental conditions.

**Supplementary Information:**

The online version contains supplementary material available at 10.1038/s41598-025-86018-7.

## Introduction

Peas are an important protein crop as well for human as for animal nutrition well adapted to cool climatic conditions. As leguminous crop, they simultaneously provide important ecological services such as biological N fixation. However, frequently pea production is limited by multiple soil-borne pathogens that cause pre- and postemergence death, wilts and foot and root rots which are collectively referred to as a ‘pea root rot pathogen complex’^1^.

The diversity and predominance of pathogens in the root rot complex can vary greatly depending on the pedo-climatic conditions, cropping history, geographical location, year and even between years within the same location^2–6^. Thus, in Germany, surveys from 2005 to 2007 in conventional spring peas^7^ and 2009–2012 in organic spring peas^8^ showed that the primary constituents of the pea root rot pathogen complex were the species *Didymella pinodella* (syns. *Phoma medicagins*, *Phoma pinodella*,* Peyronellaea pinodella*) typically accompanied by a moderate presence of *Fusarium redolens*, *F. avenaceum*, and the members of the *F. solani* (FSSC) and *F. oxysporum* (FOSC) species complexes. In contrast, in Canada, the pathogen complex is primarily characterized by the dominance of *Aphanomyces eutheices* and *F. avenaceum*^5^, whereas in the USA, *F. avenaceum* and FOSC are the most frequently reported^9^. Recent studies in France and the UK identified *D. pinodella*^10^ and the FOSC, FSSC and *F. redolens*^11,12^ as the predominant species within the pea root rot complex. Understanding the factors that shape the pea root rot complex pathogen community and their influence on yield is essential for effective disease risk assessment and disease management.

While peas are the most widely grown grain legume crop in Germany^13^, pea production in Germany sharply declined by 75% from 163.610 hectares (ha) in 2001 to about 42.000 ha in 2014^13^. Low and instable yields together with high susceptibility to soil-borne pathogens had been discouraging farmers from growing this crop. With the implementation of the Germany protein crop strategy in 2012^14^ and the EU Common Agricultural Policy (CAP) greening measures in 2015, the trend was reversed and by 2022, pea production was about 107.000 ha. As organic pea production overall has remained relatively stable during the past 20 years^15^, this increase is primarily due to conventional farmers that are now integrating mostly spring pea into their rotations. Often, these farmers are growing legumes for the first time after more than 10 years, providing a unique opportunity to study the effects of cropping history on pea health. For this, from 2016 to 2019 we conducted yearly surveys on pea root health in conventional and organic peas, to evaluate the impact of farming system (organic versus conventional), pedo-climatic conditions and crop rotation management on root health and root rot pathogens and their influence on pea yield.

The specific objectives of the study were to (1) determine the current root health status and examine the identity and prevalence of root rot pathogens associated with spring and winter peas in Germany, (2) determine the effect of cropping systems on root health as well as the diversity of *Fusarium* and *Didymella* communities in pea roots and, (3) relate the changes in pea root health, cropping history and pedo-climatic conditions to the variations in the *Fusarium* and *Didymella *communities and pea yield. Additionally, (4) we present findings on the genetic variability of the FOSC and FSSC isolates recovered, and compare results from this study with the outcomes of a parallel survey on faba bean root rot in Germany^6^, in order to contribute to a better understanding of root health and pathogen dynamics in these two major protein crops.

## Results

### Environmental and soil conditions of the sampled fields

The 133 organic and conventional pea fields sampled represented a wide range of environments with respect to climatic and soil conditions (Tables 1 and 2). Sowing conditions ranged from very wet (up to 71 mm of rainfall in the 2 weeks preceding sowing) to completely dry, as well as from very cold (with a minimal mean temperature of −4.9 °C two weeks before sowing) to warm conditions (a maximum mean of 13.8 °C before sowing). Similarly, the conditions during plant emergence varied greatly, with up to 72 mm of rainfall two weeks after sowing to entirely dry periods (Table 1). For spring pea, the driest conditions were recorded in 2018, where a field received as little as 21 mm of rainfall from sowing to sampling. Wettest conditions were observed in 2016, with a spring pea field receiving a 533 mm of rainfall over the same period. In winter peas, the driest conditions were recorded 2016/2017, with a field receiving only 163 mm of rain during the growing season, while the wettest conditions occurred in 2018/2019, where a field received 1001 mm of rainfall from sowing to sampling (Table 1).

**Table 1:** Variability in pedo-climatic conditions across spring and winter pea fields in organic and conventional farming systems during the 2016–2019 sampling period.

Parameter	Organic spring peas (*n* = 23)^1^			Conventional spring peas (*n* = 76)			Organic winter peas (*n* = 29)			Conventional winter peas (*n* = 5)		
	Min	Max	Median	Min	Max	Median	Min	Max	Median	Min	Max	Median
Soil												
% SOM	1.1	3.6	2.1	1.3	4.4	2.6	2	4.2	2.7	2.2	5.1	3.1
% sand	9	86	59	2	77	30	7	40	16	10	67	24
% silt	10	68	27	12	75	51	38	75	61	22	40	32
% clay	4	39	15	5	36	20	15	36	23	11	50	38
pH	5.8	7.3	6.4	5.6	7.5	6.7	5.8	7.5	6.6	6.4	7.5	7.2
Water												
ppt mm (14 days prior to sowing)	2	51	21	0	71	14	0	59	18	19	25	22
ppt mm (14 days after sowing)	5	40	16	0	72	26	2	57	22	0	33	8
ppt mm (sowing-sampling)	29	533	146	21	365	150	205	1001	380	163	385	317
Temperature												
Average temp, ^o^C (01.Jan-sowing)	0.6	5.2	2.8	0.1	5.2	2.6	2	9.2	5.6	−0.8	7.5	5
Number of days < 0° in March	0	9	0	0	12	0	0	11	0	0	5	0
Average temp, ^o^C (sowing − 14 days)	2.2	13.7	7.9	−4.9	13.3	6.5	7.2	16.2	11.8	4.6	13.3	12.1
Average temp, ^o^C (sowing + 14 days)	5.8	17.8	8.8	3.1	15	8.4	4.5	13.5	9.1	7.6	13.6	10.1
Average temp, ^o^C (sowing-sampling)	10.9	18.5	13	3.8	17.2	12.3	3.9	8.2	5.8	5.2	11	7.2
Temp, sum ^o^C (sowing-sampling)	798	1284	1060	773	1462	1071	843	2227.2	1390	981	1774	1461
Cropping history												
# Cereals (5 year history)^2^	0	5	4	0	5	3	2	5	3	1	4	3
# Grain legumes (5 year history)^3^	0	2	0	0	2	0	0	5	1	0	1	0
Years since last grain legume crop^4^	2	≥ 11	≥ 11	0	≥ 11	≥ 11	1	≥ 11	5	3	≥ 11	≥ 11

^1^n=number of sampled fields: organic systems 23 spring and 29 winter pea; conventional systems 76 spring and 5 winter pea fields.

^2^Number of cereals grown during the 5 years preceding the sampling.

^3^Number of times grain legumes were grown during the five years preceding the sampling.

^4^Number of years since a grain legume crop was grown in the field prior to the sampling. No data beyond 11 years were available.

**Table 2:** Soil clusters formed by grouping fields based on their similarity in soil abiotic properties, along with the number of organic and conventional spring and winter pea fields and roots sampled in each year.

System	Pea type	Cluster	Year^1^				*N* ^2^	*N* ^3^	pH^4^	SOM (%)	Sand (%)	Silt (%)	Clay (%)
			2016	2017	2018	2019							
Organic	Spring	1	2	4	6	3	15	299	6.3	1.8	67.2	22.2	10.6
		2	2	1	0	1	4	78	6.5	2.7	28.2	50.7	21.1
		3	1	1	0	2	4	64	7.0	3.1	21.3	50.8	27.9
	Winter	2	3	8	6	4	21	380	6.5	2.6	18.4	59.6	22.1
		3	1	2	1	4	8	135	7.1	3.3	17.6	55.4	27.0
Conventional	Spring	1	3	6	6	4	19	375	6.5	2.5	62.3	25.9	11.8
		2	11	10	7	7	35	690	6.5	2.6	23.1	56.5	20.4
		3	5	5	6	6	22	439	7.1	3.0	20.5	51.5	28.0
	Winter	1	0	1	0	1	2	40	6.7	2.3	62.7	24.9	12.4
		3	0	1	1	1	3	60	7.3	3.9	18.9	37.3	43.7

^1^Number of fields sampled in 2016, 2017, 2018 and 2019.

^2^N= Total number of fields.

^3^n = Number of roots evaluated.

^4^Mean value of soil abiotic properties.

Soil types ranged from light sandy soil (up to 86% sand) to heavy clay soils (up to 50% clay) with pH levels ranging from 5.6 to 7.5 and a high variation in soil organic matter (SOM) content from only 1.1% to up to 5.1% (Table 1). The sampled fields grouped into 3 clusters based on their similarities in soil pH, sand, silt, clay and organic matter content. The first two dimensions of the Hierarchical Clustering on Principal Components (HCPC) analysis accounted for 77% of the variance in the dataset, 57% in the first dimension that separated sand dominated fields (Cluster I) from silty-clay soils in Clusters II and III (Table 2). The second dimension explained an additional 20% of the variance and showed the strongest association with SOM content.

Cluster I comprised 15 organic and 19 conventional spring pea fields and 2 conventional winter pea fields with sandy soils and a pH of around 6.4. The mean SOM content in Cluster I was 1.8% in organic fields and approximately 2.5% in conventional fields. The largest Cluster II comprised silty-clay soils with mean pH of 6.5 and 2.6% SOM, represented by 39 spring pea fields, 4 of organic and 21 out of the 29 organic winter pea fields. Cluster III soils were again silty-clay but with higher pH (about 7.1) and mean SOM contents (3.0 for spring pea and 3.5 for winter pea fields). Four of the 26 spring pea fields and 8 of the 11 winter pea fields were organic (Table 2).

### Crop rotations

**Fig. 1:**
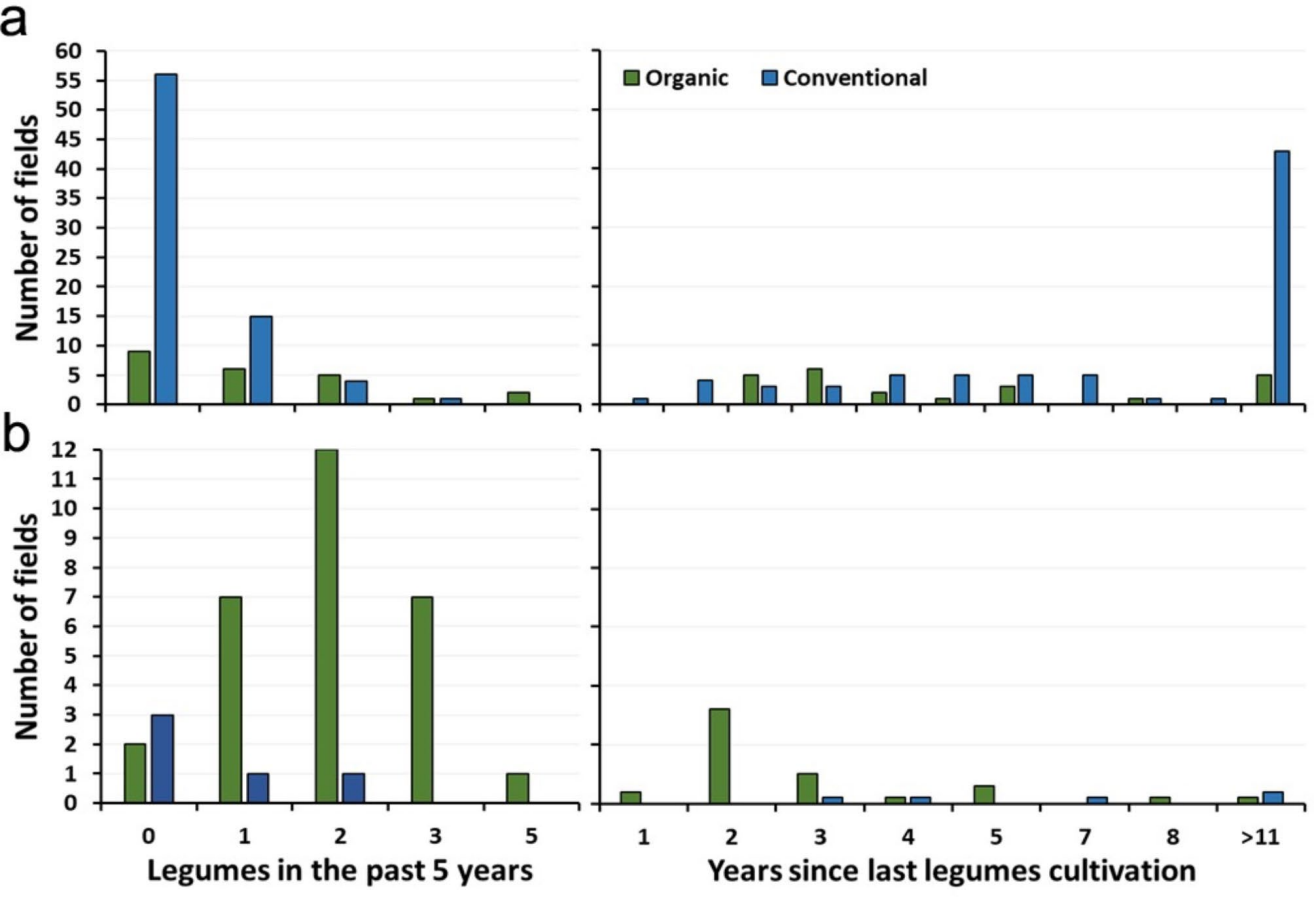
Legumes in organic and conventional crop rotations. (**A**) Spring pea and (**B**) Winter pea: On the left, the number of fields with legumes grown in the past five years prior to sampling; on the right, the number of fields and the time since legumes were last cultivated before sampling. Data for more than 11 years were not available.

The proportion of legumes in the five years preceding pea sampling varied considerably depending on system and pea type. In 14 out of the 23 organic spring and 27 out of the 29 organic winter pea fields legumes had been cultivated in the five years preceding our sampling (Fig. 1). In contrast, out of the 81 conventional fields sampled, 59 had not been cropped to any legume in that period.

In half (7) organic spring pea fields with legumes in the past five years, grain legumes had been cultivated once (four times pea and two times faba bean and once, pea and faba bean). Clover and alfalfa were grown on 13 fields either in one out of 5 years (8 fields), or for two (3 fields) or three (2 fields) consecutive years. All except one organic farms grew cereals, either in one (3 fields), two (2 fields), three (4 fields), four (9 fields) or five (4 fields) years during the preceding 5-years. In addition, eight out of the 23 organic spring pea fields grew this crop in mixture with oats (*Avena sativa*; 5 fields), false flax (*Camelina sativa*; 2 fields) or spring wheat (*Triticum aestivum*; 1 field) (Supplementary Table S1).

Grain legumes were present once (9 times), twice (5 times) and trice (once) during the five years before organic winter peas. Out of these 15 cases, 11 fields had been planted with pea, 9 once and 2 twice; faba beans were grown in 4 fields once. Additionally, clover and alfalfa had been grown on 17 fields, either for one (10 fields) or two years (7 fields). Cereals had been grown in all 29 fields: 9 times twice and 9 times trice, 10 times for four years, and once in all five years before winter peas. All organic winter peas were grown in mixtures with triticale (19 fields), rye (9 fields) of winter wheat (1 field) (Supplementary Table S1).

Among the 20 conventional spring pea fields with legumes in the previous five years, 11 had been cultivated with pea once and two twice and one with faba bean. In one of the winter pea fields two years grass-clover and in one faba beans had been grown. Five farmers also had clover and alfalfa based mixtures in the rotations. The conventional 5-year rotation plans included two (14 fields), three (29 fields), four (23 fields) or five (5 fields) years of cereals. Only two conventional fields had not at all been sown to cereals (Fig. 1). Conventional peas were predominantly cultivated as pure stand (73 fields), whereas three spring pea fields were grown in mixture either with barley (2 fields) or false flax (1 field). Among the 5 conventional winter pea fields, two farmers grew the crop in mixture with triticale (Supplementary Table S1).

### Field level root rot incidence and root health status

Overall, a total of 2560 pea roots were assessed for the severity of root rot symptoms. In spring pea fields (total number of roots assessed, *n* = 1945), root heath status ranged from completely healthy (disease severity rating DSR = 1; absence of any symptoms) to moderately diseased (DSR = 6.1 in organic fields, *n* = 441 and; DSR = 6.2 in conventional fields, *n* = 1504). Mean DSR in organic peas (3.0) was significantly higher (*P* = 0.04) than in conventional peas (2.2). Clearly visible symptoms of field level root rot with mean DSR > 3 were recorded in 7 (30%) out of 23 organic and 15 (20%) with no statistically significant year or systems effects (Table 3). Root rot was equally severe in the 15 organic spring pea pure stands and the 8 organic species mixtures (Supplementary Table S2).

**Table 3:** Number of fields with field level root rot incidence (disease severity ratings > 3) and mean root rot disease severity ratings (DSR) for organic and conventional pea fields sampled across Germany from 2016 to 2019.

	Sampling year	Organic				Conventional			
		2016	2017	2018	2019	2016	2017	2018	2019
Spring pea	Field level root rot incidence^1^	2/5	2/6	3/6	0/6	3/19	3/21	5/19	4/17
	Mean root rot severity^2^	4.2	4.8	5.6	-	3.9	4.8	3.5	4.1
	n^3^	88	115	120	118	371	416	379	338
Winter pea	Field level root rot incidence	0/4	2/10	0/7	0/8	n/a	0/2	0/1	0/2
	Mean root rot severity	-	4.9	-	-	n/a	-	-	-
	n	29	190	136	160	n/a	40	20	40

^1^The number of organic and conventional fields with clearly visible symptoms of root rot i.e. number of fields with mean disease severity rating (DSR) > 3/the total number of fields sampled in the respective year.

^2^Mean root rot severity for fields which had mean disease severity rating greater than 3. For overall means, see text.

^3^n: total number of roots evaluated for severity of root rot symptoms.

’-‘ no fields within the category; n/a – no conventional winter pea fields were sampled in 2016.

There was no significant year effect on spring pea root rot incidence on the field level (*P* = 0.42) or mean disease severity ratings (*P* = 0.18) in data analyzed across the management systems (the Krusal-Wallis test). Data from winter pea surveys were not subjected to the analysis due to heathy appearance of plants.

Winter peas appeared mostly healthy with overall mean DSR of 2.0 and 1.5 in organic (*N* = 29; *n* = 515) and conventional (*N* = 5; *n* = 100) fields, respectively. Moderate field level root rot was recorded only on two organic winter pea fields (7%) sampled in 2017 with mean DSR of 4.3 and 5.5 (Table 3).

### *Fusarium* and *Didymella* species associated with pea roots

A total of 5097 isolates representing 4 *Didymella* and 14 *Fusarium* spp. were recovered from 2651 roots used for the fungal isolations over the 4-yr study period (Supplementary Table S1). With 56% of all roots infected, *D. pinodella* was the most frequently recovered species, followed by *F. redolens*, *F. avenaceum* and the members of the FOSC with approximately 27% infected roots. Members of the FSSC (18% roots infected) and the species *F. tricinctum* (14%) were the next most frequently isolated. *Fusarium equiseti* and *F. culmorum* were found with overall root infection rates of about 5%. Also found but represented with few isolates only were the species *F. acuminatum*,* F. graminearum*,* F. sporotrichioides*,* F. crookwellense*,* F. torulosum*,* F. flocciferum*,* F. sambucinum*,* D. pinodes*,* D. lethalis and D. eupyrena (*syn. *Juxtiphoma eupyrena).*

**Fig. 2:**
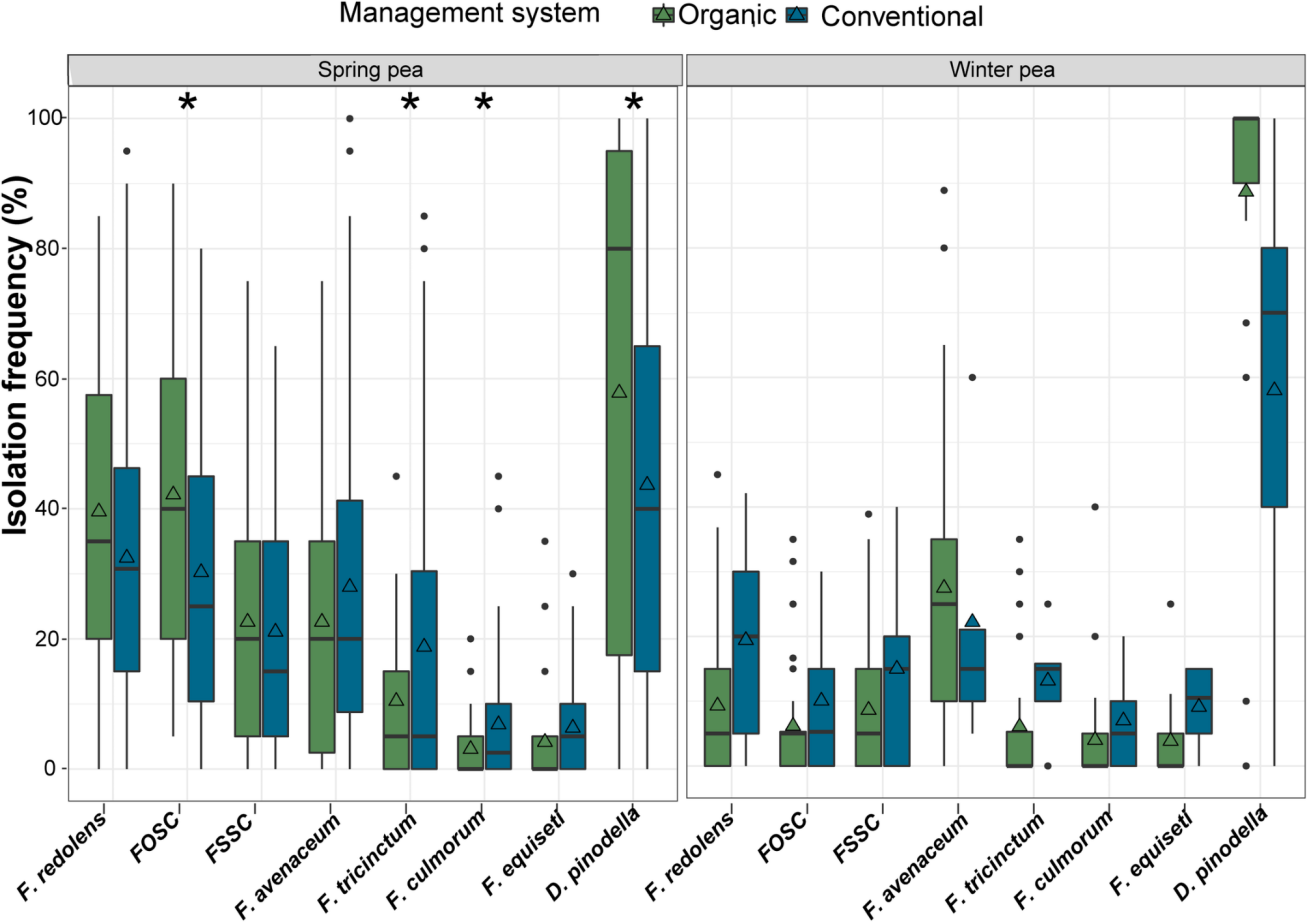
Effect of management system on isolation frequency (%) of the top eight fungal species recovered from spring and winter pea roots. Asterisks indicate significant differences (*P* < 0.05) between organic and conventional fields for each fungal species separately (Sidak-adjusted pairwise least significant means comparisons). The horizontal line in the boxplot shows the median value, the bottom and tops of the box the 25th and 75th percentiles and the vertical lines the minimum and maximum values, outliers as single points. Mean values are marked with triangles.

Isolation frequencies varied significantly depending on the fungal species (*P* < 0.0001), pea type (spring vs. winter, *P* = 0.002) and year (*P* = 0.005) with significant interactions between fungal species isolation frequencies and pea type, growing system and year (*P* < 0.0001). Organic spring pea roots were infected more frequently with *D. pinodella* (*P* = 0.005) and members of the FOSC (*P* = 0.003) compared to conventional spring peas (Fig. 2). The higher overall isolation frequencies of *D. pinodella* in organic systems compared to conventional were mainly due to higher frequencies of this species in 2016 and 2017 in organic fields compared to the conventional (ca. 70% infected roots in organic systems vs. ca. 40% infected roots in conventional systems). In 2018 and 2019, the species showed similar frequencies in both management systems (ca. 30 and 60% roots infected in 2018 and 2019, respectively). The FOSC members were consistently 10–20% more frequent in roots collected from organic (35–51%) compared to conventional systems (28–35%), but these differences were not statistically significant (Supplementary Table S3). In contrast to FOSC and *D. pinodella*, organic spring pea roots were less frequently infected with *F. tricinctum* (*P* = 0.0006) and *F. culmorum* (*P* = 0.006) compared to conventional although at overall low frequencies. Isolation frequencies of *F. redolens* (*P* = 0.08), the FSSC (*P* = 0.6), *F. avenaceum* (*P* = 0.01) and *F. equiseti* (*P* = 0.01) varied somewhat among years with no statistically significant effect of management system (Fig. 2 and Supplementary Table S3). None of the isolation frequencies of the individual fungal species was affected by species mixtures in organic spring peas (Supplementary Table S2).

On average, 89% (72–98%) of the organic winter pea roots were infected with *D. pinodella*. Infections with *Fusarium* spp. were considerably lower (Fig. 2 and Supplementary Table S3) with *F. avenaceum* most frequently recovered (27% overall mean isolation frequencies), followed by *F. redolens* and the FSSC (9%). The remaining *Fusarium* species occurred overall at frequencies of 6% and lower (Fig. 2 and Supplementary Table S3). Conventional winter pea roots followed a similar trend with *D. pinodella* (58%) predominant, followed by *F. avenaceum* (22%) and *F. redolens* (19%) (these data were not included in any statistical analysis due to very small sample size (*N* = 5)) (Fig. 2 and Supplementary Table S3).

### Phylogeny

**Fig. 3:**
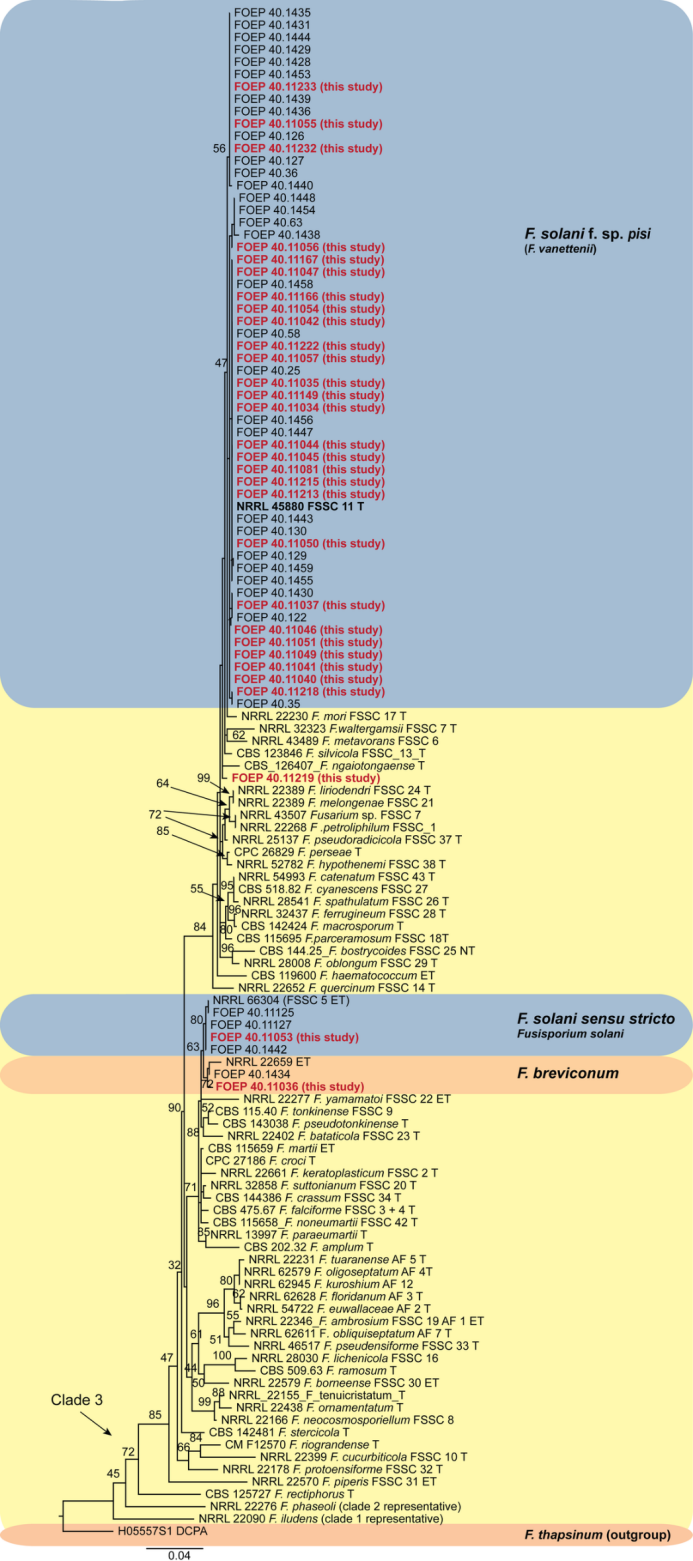
The maximum likelihood (RAxML) tree constructed using partial *tef1* alpha gene sequences from 30 isolates of the *Fusarium solani* species complex (FSSC) examined in this study highlighted in bold and red (i.e. FOEP-this study). FOSC isolates from parallel previous study^6^ recovered from faba bean were also included in the analysis were also included in the analysis (FOEP isolates). Epi- and ex-type strains are marked with a ‘T’^53,69^. The scale bar represents 0.04 expected changes per site, and the tree is rooted with *F. thapsinum* (H05557S1 DCPA).

Based on the single-locus phylogeny the 30 FSSC isolates belonged to three different lineages, all nested within clade 3 (Fig. 3). The majority of the isolates (28 out of 30) closely matched *Fusarium vanettenii (*syn. *F. pisi*, *F. solani* f. sp. *pisi*). In addition, one isolate was placed within the *F. solani* sensu stricto lineage, and one isolate matched *F. breviconum*.

**Fig. 4:**
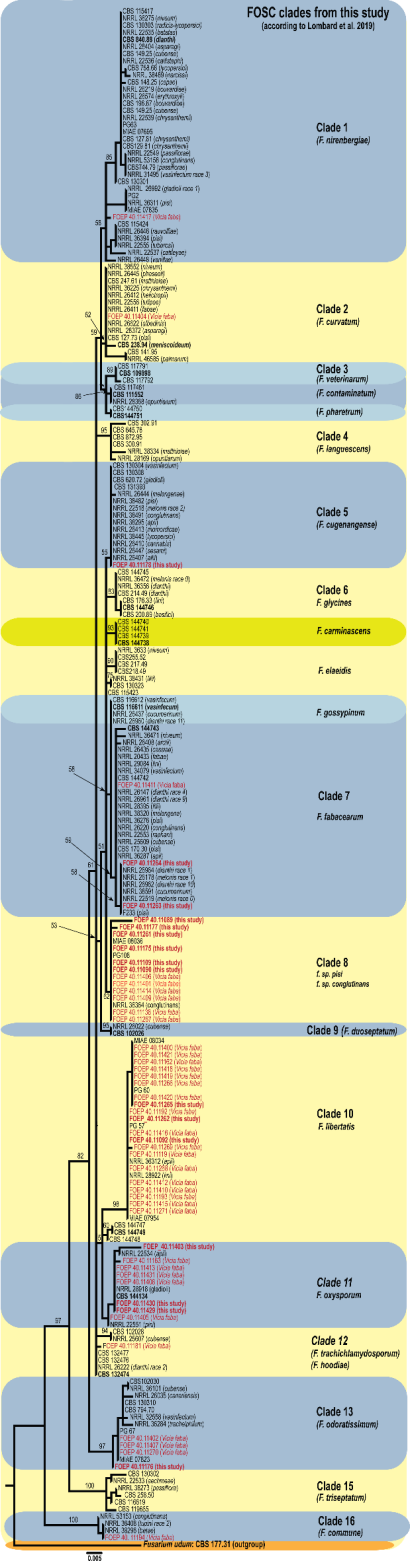
The maximum likelihood (RAxML) tree constructed based on partial *tef1* alpha gene sequences from isolates of the *Fusarium oxysporum*species complex (FOSC) used in this study, highlighted in bold and red (e.g., FOEP-this study). The FOSC isolates from parallel previous study^6^ recovered from faba bean were also included in the analysis and are indicated in red with host in brackets. Along with the FOSC clades are given proposed species names in the FOSC along with epi- and ex-type strains (in bold) suggested by Lombard et al.^16^. The scale bar represents 0.005 expected changes per site, and the tree is rooted with *F. udum* (CBS 177.31).

The 17 FOSC isolates were nested within six clades (Fig. 4). The largest group consisting of 6 isolates (clade 8 in this study) showed the closest genetic relationship to the previously classified *F. oxysporum* forma specialis (f. sp.) *pisi* (PG108; MIAE 08036) and f. sp. *conglutinans* (NRRL 36364). Two isolates were associated with clade 7, described as the *F. fabacearum* and *F. gossypinum* in Lombard et al. (2019) which were not entirely resolved in our single-locus analysis. In addition, individual isolates were nested within clades 5 and 13 corresponding to the previously described *F. cugenangense* and *F. odoratissimum* (Lombard et al., 2019).

### The relationship between pathogen occurrence, root health, yield and environmental factors

Cool temperatures in early spring favored *F. redolens* in organic winter peas and both, organic and conventional spring peas were favored by cool temperatures in early spring. In addition, in spring peas, the species was overall reduced in wetter years, and only in organic systems if more grain legumes occurred in the rotation (Table 4).

**Table 4:**
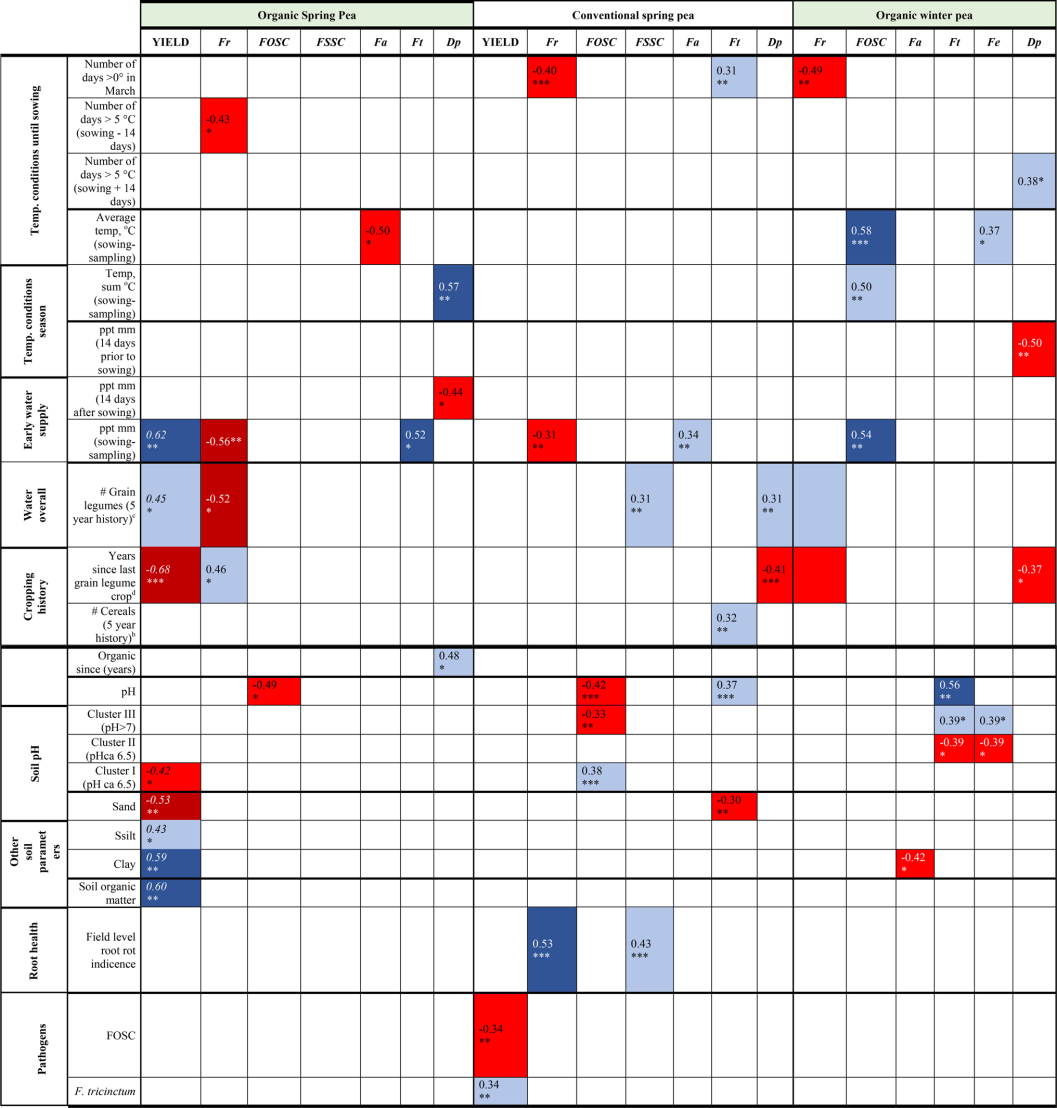
Summary of Pearson correlation analysis results, demonstrating key environmental and cropping history factors impacting pea yield and the abundance (isolation frequencies) of major fungal species in the roots of organically (org.) and conventionally (conv.) cultivated spring and winter pea. Positive correlations are indicated with ‘+’ and indicate increase in the species isolation frequencies (root colonization rates). Negative correlations are indicated with ‘-’ and indicate decrease in the species isolation frequencies (root colonization rates). ^1^+/- early cold: positive (+) or negative (-) correlation with Average temp. °C (Jan-sowing) and/or, Number of days < 5°C (14 days prior to or after sowing-sowing) and/or, Number of days < 0° in March and/or, Average temp. °C (14 days prior to sowing-sowing) and/or, Number of days < 5°C (sowing-14 days after). ^2^+ warm season: positive correlation with Average temp. or temp. sum °C (sowing-root sampling); +cool season: negative correlation with Average temp. or temp. sum °C (sowing-root sampling). ^3^+/- early wet: positive (+) or negative (-) correlation with Precipitation sum (mm) (28 days before sowing to sowing) and/or, Precipitation sum (mm) (sowing to14 days after).^4^+dry season: negative correlation with Precipitation sum (mm) (sowing-root sampling).

The members of the FOSC in organic and conventional spring peas were negatively correlated with pH and in conventional systems with cluster III soils while cluster I soils favored their occurrence. In contrast, in organic winter peas overall warm temperatures and wet conditions correlated with FOSC occurrence. The isolation frequencies of the FSSC members correlated positively only with the frequency of grain legumes in the rotation under conventional conditions. No significant effects of environmental conditions in organic systems could be found (Table 4).

Warmer temperatures during the season affected *F. avenaceum* in organic spring peas negatively while in conventional spring peas, it was favored by higher humidity. In organic winter peas this species correlated negatively with the clay content of the soil (Table 4).

In conventional spring peas, *F. tricinctum* was positively correlated with warm temperatures before sowing, cereals in the rotation and the soil pH and sand contents. The positive association with pH was also observed in winter peas. In organic spring peas, it only correlated with wetter conditions during the season. In spring peas *F. equiseti* was not affected by environmental conditions while in organic winter peas the species correlated positively with warmer temperatures during the season (Table 4).

The main factor correlating with increased *D. pinodella* in all growing systems was the cropping history. Both, in conventional spring peas and organic winter peas, the longer the break since the last grain legumes were grown the lower the frequency of *D. pinodella* isolations. In organic spring peas, instead, the species correlated with the years since conversion to organic which in fact also is an indicator of legume frequency in the system. In organic spring peas, an overall warm season enhanced the frequency while good water supply after sowing was associated with reduced infections. In contrast, in winter peas warm conditions with good water supply around sowing reduced *D. pinodella* root infection rates (Table 4).

Except for *F. redolens* and the FSSC complex in the conventional spring peas none of the fungal species identified correlated with field level root rot incidence, i.e. disease severity > 3 (Table 4).

Water availability, cropping history and soil properties correlated with organic spring pea yields in various ways but not for conventional spring peas or organic winter peas (Table 4). Only conventional yields appeared to be weakly affected by the frequencies of root associated *Fusarium* and *Didymella* spp. but in part with contradictory trends. Correlations were negative with the FOSC (*r* = −0.34) complex but positive with *F. tricinctum* (*r* = 0.34) (Table 4).

## Discussion

Pea roots appeared mostly healthy irrespective of the highly variable pedo-climatic conditions and rotational histories of the 128 organic and conventional spring and winter pea fields sampled. Despite this, 14 *Fusarium* spp. and 4 *Didymella* spp. were isolated, including *D. lethalis* and *F. flocciferum*which were identified for the first time in Germany^17,18^. *Didymella pinodella* was the predominant species, followed by *F. redolens*, *F. avenaceum* and members of the *Fusarium oxysporum *species complex (FOSC). These findings agree with previous reports from Germany which also found similar spectra of these potentially pathogenic fungi in symptomatic pea roots^7,19^ and predominantly asymptomatic faba bean^6^ roots. Winter pea roots in particular appeared healthy. These cultivars typically have a higher tannin content than spring peas^19^. This may have contributed to the higher resistance and absence of symptom expression which makes them particularly attractive to organic farmers. In contrast, most winter pea varieties are not attractive to conventional farmers due to their indeterminate growth which requires a support crop and poses technical challenges in planting and harvesting including the low economic value of the crop. Consequently, all winter pea farmers included in this study cultivated this crop in mixtures and they were predominantly organic.

Phylogenetic analysis inferred from the *tef1*gene sequences placed the 17 FOSC isolates in 6 clades, all previously associated with pea and/or faba bean roots. This high phylogenetic diversity observed has been reported in other studies^6,11,12,20^ and likely is a result of the polyphyletic origin of different *F. oxysporum*formae speciales^21^. Additional analyses are needed to determine the role of these isolates in the pea root rot complex. This is particularly important as the FOSC members also includes endophytes with non-pathogenic characteristics. Also, the characteristics of the different FOSC isolates found in the same host plant but belong to different genetic lineages are not well understood. These isolates/lineages may potentially show high variation in aggressiveness or specific cultivar-pathogen interactions.

In contrast, the FSSC isolates were phylogenetically less diverse than FOSC, with most belonging to the *Fusarium vanettenii* lineage (syn. *F. pisi*, *F. solani* f. sp. *pisi*). One isolate was placed within the *F. solani* sensu stricto lineage, and one isolate matched *F. breviconum*. These results are consistent with previous studies which also reported similar phylogenetic diversity including the broader host range for the single FSSC isolates recovered from roots of several legumes including pea, faba bean, subterranean clover, white clover and winter vetch^6,11,12,22^.

Higher levels of root rot symptoms in conventional spring pea fields correlated with higher isolation frequencies of *F. redolens* and the FSSC. In contrast, in organic systems field level root rot incidence was recorded in approximately 30% of the fields but could not be linked to any of the major fungal species isolated. Under organic conditions, the lack of correlation between root health parameters (i.e. visible damage) and fungal species specific isolation frequencies as well as between these parameters and yield, could be due to the stronger impacts of soil conditions as inputs are severely limited. In addition, factors not evaluated in this study such as weed infestation, insect populations and other physicochemical soil conditions can overshadow the direct influence of root-health related factors on yields and infection rates of potentially pathogenic and other root-associated fungi.

While management system (organic vs. conventional) or pea type (spring vs. winter) did not affect the spectrum of fungal species isolated, differences in isolation frequencies were present especially for *D. pinodella.* This can be explained mostly by the overall lower frequency of legumes in the conventional cropping histories as repeated grain legume cropping has been shown to result in increased *D. pinodella* abundance in soil and roots of pea and faba bean^2,6^. The predominance of *D. pinodella* over *Fusarium *spp. in organic winter pea roots also agrees with previous results^19^. The high root infection rates by *D. pinodella* in organic winter peas compared to organic spring peas further suggest an ecological advantage of this species in cooler and moist environments compared to *Fusarium* spp. Infection success of *D. pinodella* is especially high directly after sowing and quickly declines within a few days especially in the presence of beneficial microorganisms such as *F. equiseti*^23^. It is also possible that the generally higher tannin content in winter peas along with variations in root exudation played a role in modulating the plant-associated microbiome and suppressing *Fusarium* infections.

In both, organic and conventional systems FOSC isolation frequencies negatively correlated with soil pH a fact that has been repeatedly reported^6,24–26^. As organic fields were primarily characterized by sand dominated low SOM soils (Cluster I) and lower pH levels while in conventional systems, silty soils (Cluster II) predominated (Table 2), it is likely that the abundance of FOSC in organically grown spring peas is at least partly due to the soil characteristics. Lower SOM and soil pH levels typically are characteristic of sandy soils. Such soils mostly have reduced water retention capacity resulting in lower yields of both peas (as observed in this study) and faba beans^6^ as yield of both crops highly depend on soil water availability. With yields already impeded in the sandy low SOM fields, the impact of FOSC on yield may not have been distinguishable any more in contrast to its impacts in the better conventional soils.

The cereal dominated conventional field histories resulted in higher isolation frequencies of *F. tricintum* and likely contributed to increased frequencies of *F. culmorum* in conventional spring pea roots compared to organic. *Fusarium tricintum* also correlated positively with soil pH and negatively with sand. The two species are typical members of the *Fusarium* complex associated with ear, stem and root rots in various small-grain cereals and maize and are responsible for pre-harvest mycotoxin contamination^27,28^. However, these species are usually of minor importance in the pea root rot complex and their roles are not fully understood. Thus, conventional spring pea yields actually correlated positively with the abundance of *F. tricintum *in roots, a relationship we also found in faba beans^6^. In contrast, *F. tricinctum* has been to be reported potentially important pathogen of soybeans^29^. *Fusarium culmorum *is often implicated as a weakly to moderately aggressive pea root rot pathogen^30,31^.

The wide spread occurrence of *F. redolens*,* F. avenaceum*,* F. equiseti* and the members of the FSSC in spring peas in both management systems over a range of soil and environmental conditions indicates their good adaptation to diverse pedo-climatic conditions. With the exception of *F. equiseti* which has been shown to contribute to disease reduction in various crops^32,33^ including pea root rot^23^, all of the remaining *Fusarium* species are a major part of the pea root rot complex across different climatic and soil conditions, including Canada, France, USA and Germany.

Consistent with recent results in spring faba bean^6^, abundance of *F. redolens* in spring pea roots correlated with cold conditions early in the season during sowing and plant emergence followed by a dry growing season. This highlights the importance of abiotic plant stressors in enhancing the colonization process by this potential pathogen, likely contributing to the higher abundance of this species in conventional pea roots with clear symptoms of root rot. Interestingly, in organic spring peas *F. redolens* correlated negatively with frequency of grain legumes in the rotation. This is in contrast to recent reports from Canada^2^ and Germany^6^ where increased abundance of this fungus positively correlated with grain legume-intensive rotations. These differences could be due to a combination of factors specific to this study, including the influence of prior legume crops and/or other soil and agronomic practices. These factors may have shaped the pea root rot complex community potentially favoring accumulation of more specialized fungal species like *D. pinodella* at the expense of *F. redolens*. It is also possible that specific soil suppressiveness against *F. redolens* that depends on the regular cropping of grain legume species played a role. A more in-depth microbial community analysis could help elucidate the interaction of cropping system and the broader microbial community structure in influencing the symptom expression and the presence or suppression of single pea pathogens. We also cannot exclude the possibility of the isolation procedure contributing to these results. The choice of agar medium and the inherent challenges of culture-based methods to recover specific fungal species have been reported previously^5,34,35^. To overcome these limitations DNA-based detection techniques like quantitative real-time (q)PCR assays or next generation sequencing (NGS) could be employed. However, qPCR assays targeting all major fungal species identified in this study have become available only after the start of this research^34,36^. Additionally, the application of NGS was beyond the scope and focus of this study, which was primarily focused on examining the occurrence of *Fusarium* and *Didymella* species, their genetic diversity and interactions with cropping systems and yield.

The positive correlations of *F. avenaceum* with cold seasons in organic spring peas and, wet seasons in conventional spring peas, suggest that abiotic plant stress enhances the colonization process by *F. avenaceum*. While this species plays a significant role in the pea root rot complex in Canada and the USA^5,9^ it is mostly an opportunistic pathogen in Germany where it has also been shown to be favored by cool and water logged conditions over winter^6,19^.

In organic systems, the FSSC frequencies were not affected by any of the pedo-climatic or rotational history characteristics tested while under conventional conditions it correlated with root rot incidence and a higher frequency of grain legumes in the rotation. This fungal complex is known to be of importance in pea root health^3,9,12,37^ and we have no explanation why it did not play a prominent role under organic conditions despite equal isolation frequencies in both systems.

Taken together, in all years, several potential pathogens could be found in predominantly asymptomatic pea roots, showing that the *Fusarium* and *Didymella* spp. associated with peas often reside in the roots without causing substantial damage^6,38–40^. The occurrence of such asymptomatic infections is likely the result^41^ of a balanced antagonism between the plants defense mechanisms and the virulence factors of the pathogens. Given that biological interactions are never neutral, we recently showed, for example, that asymptomatic infections with *D. pinodella* can reduce wheat biomass^39^ and can also cause severe pre-emergence death and post-emergence root rot in peas^10^. Asymptomatic root infections by *D. pinodella* and *F. redolens* have also been linked to reduced faba bean yields^6^, suggesting higher investment of the faba bean to maintain a balanced antagonism with these fungi. The negative correlation of conventional pea yields with members of the FOSC in this study suggests similar underlying interactions. Furthermore, environmental factors and the timing of root infections are important in maintaining balanced antagonism and influence disease development, including the expression of visible disease symptoms^6^. *Fusarium* and *Didymella* spp. are often opportunistic pathogens that can cause damage especially well if pant stress occurs in early crop growth stages, whereas pea can tolerate well root infections in later growth stages^23^ if environmental conditions are not too extreme (e.g. prolonged drought or rainy period). Furthermore, beneficial fungi in the roots may also have played a role in the lack of clear disease symptoms and the weak association between root rot severity and the major fungal species identified in this study e.g. beneficial *F. equiseti*^23^ and arbuscular mycorrhizal fungi^42,43^ which have the ability to manipulate plant defense and/or pathogen infection sites.

Thus, pedo-climatic factors appeared to be the main drivers for the occurrence of the most common fungal species with clear differences between spring and winter grown peas. The most obvious interactions occurred with soil pH which interacted with the occurrence of certain fungi, especially the FOSC members and *F. tricinctum* that correlated with reduced or increased soil pH values, respectively. The interactions with cropping history varied depending on the fungal species. Higher frequency of legumes in rotation or shorter intervals between legumes was associated with the presence of *D. pinodella* and to a lesser extent the FSSC. However, for *F. redolens* in organic spring peas, the opposite trend was observed. This suggests some specific microbial interactions depending on the species are involved. Only in conventional systems, root health and yields were affected by specific fungal species. Root rot incidence was associated with increased infection rates of *F. redolens* and members of the FSSC complex. In contrast, yields were negatively correlated with the frequencies of FOSC complex members and positively with *F. tricinctum*.

## Materials and methods

### Surveys, sampling and disease assessments

**Fig. 5:**
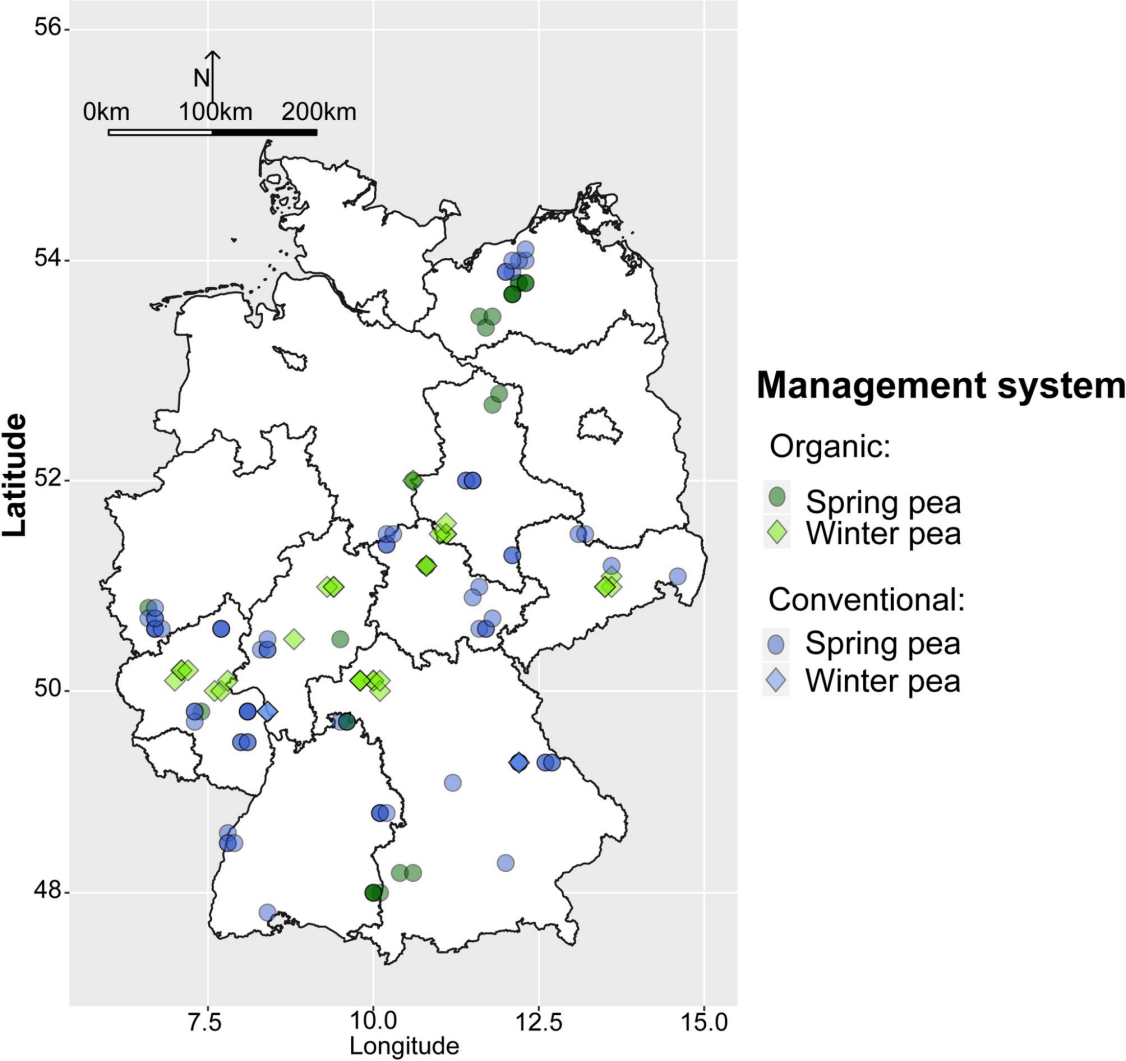
Map of Germany showing locations of the surveyed organic and conventional spring and winter pea fields.

Sample collection, fieldwork and laboratory analyses followed the procedures outlined in Šišić et al. (2022)^6^ which were used in parallel survey of faba beans. Between 2016 and 2019, 99 spring and 34 winter pea fields were sampled across Germany (Fig. 5 and Supplementary Table 1). Among the spring pea fields, 23 were managed organically and 76 conventionally. Most winter pea fields were managed organically (29 fields) and only 5 conventionally. Historical cropping data were collected directly from farmers. These included number of times (in years) that fields were planted to various leguminous species (pea, faba bean, lentil, lupin, soybean, clovers and alfalfa, vetch and the unspecified group of ‘other grain’ or ‘small seeded legumes’) and to cereals (aggregated across all cereal types) for 5 and 11 years preceding the sampling. Spring peas in 8 organic and 3 conventional fields as well as all organic winter peas and two conventional winter pea crops were grown in mixtures with cereals or false flax (*Camelina sativa*) (Supplementary Table S1).

Soil samples were collected in spring from a 0–20 cm depth by taking 20 cores from two randomly selected 5 m^2^ plots in each field, located 10 to 20 m apart. The samples were analyzed for soil pH, sand, silt, clay and soil organic matter content according to the DIN 7025:2018 − 03^44^ protocol. Meteorological data were obtained from the nearest weather stations which were always located within 10 km of the sampled fields.

**Fig. 6:**
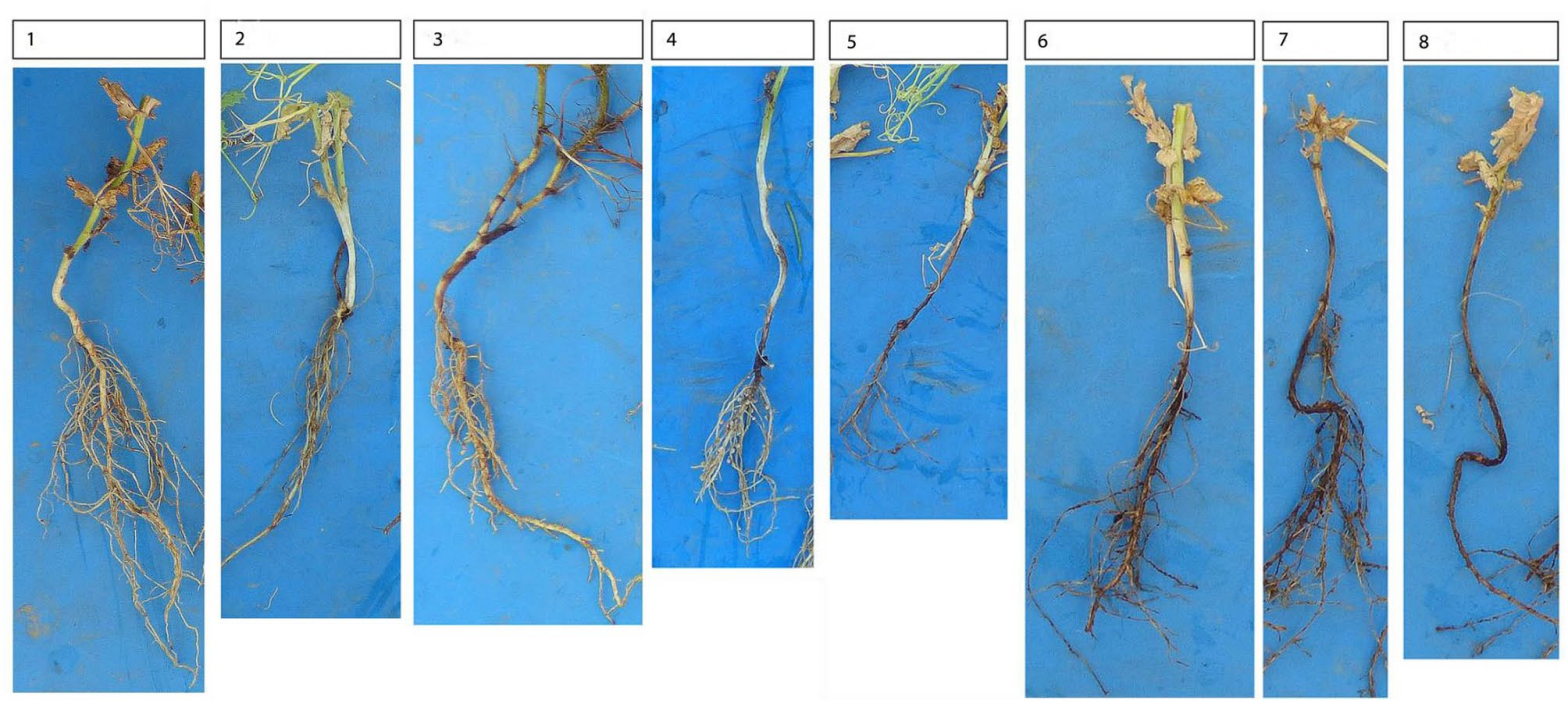
Root discoloration levels and assigned root rot disease severity ratings (1 = healthy plant to 9 = dying plant, photo credit: Lucas Langanky and Harald Schmidt).

Root sampling was performed by uprooting 36 to 40 pea plants from each field during full flowering from the same two 5 m² areas used for soil sampling. Half of the roots were immediately washed and assessed for root rot severity using a scoring system ranging from 1 to 9, where a score of 1 represented healthy plants and a score of 9 indicated dying plants^7^ (Fig. 6). The other half of the roots was sent to the University of Kassel and preserved at −18 °C until fungal isolations were performed. At pea maturity, fields were visited again and grain yields were determined by hand harvesting 2.5 m² next to the 5 m² areas used for soil and root sampling (as the initial 5 m² area had been disturbed). Gain yield was adjusted to 86% dry matter before statistical analyses.

### Fungal isolations, morphological and molecular characterization of isolates

Fungal isolations targeted species within the *Fusarium* and *Didymella *genera. Roots were first thoroughly washed in distilled water, surface sterilized using 3% sodium hypochlorite for 10 s and rinsed well in distilled water and placed on filter paper under a laminar flow hood for about 1 h to dry. From each root, three approximately 1-cm-long segments were cut out from the upper, middle and lower portions and placed on Coons media^45^ for incubation at 20˚C under a 12-hour light/dark cycle and black-light blue light. After an incubation period of 1 to 2 weeks, fungal colonies that developed from the root segments were sub-cultured separately into Petri dishes containing half-strength potato dextrose agar (19 g/l Difco PDA and 10 g/l agar). Pure cultures were generated either through hyphal tipping for *Fusarium* species or by transferring individual pycnidia for *Didymella *species. The obtained isolates were identified to the species level based on their cultural characteristics and the morphology of conidiogenous cells^46,47^.

Molecular confirmation of 124 *Fusarium* and 25 *Didymella* isolates representing 14 different fungal species was carried out by sequencing the translation-elongation factor 1 alpha (*tef1*) locus for *Fusarium* spp. and the β tubulin (*tub2*) for *Didymella* spp. (Supplementary Table S4)^48,49^. Genomic DNA was extracted from pure cultures grown on half-strength PDA (*Fusarium* spp.) and on Coons medium (*Didymella* spp.), following the method described by Doyle and Doyle (1987)^50^. The *tef1*gene was amplified using primers EF1 (5′ ATG GGT AAG GARG ACA AGA C 3′) and EF2 (5′ GGA RGT ACC AGT SAT CAT GTT 3′)^48^, and the *tub2*region was amplified with primers Btub2Fd (5′ GTB CAC CTY CAR ACC GGY CAR TG 3′) and Btub4Rd (5′ CCR GAY TGR CCR AAR ACR AAG TTG TC3′)^49^. The amplified products were visualized through electrophoresis on a 1% agarose gel and then purified using the DNA Clean & Concentrator kit (Zymo Research, Freiburg, Germany) according to the manufacturers guidelines. Sanger sequencing was conducted in both directions by Macrogen Europe Laboratories (Amsterdam, Netherlands). The raw sequence data were assembled and any errors were manually corrected using SeqMan Lasergene software (DNAStar, Madison, WI, U.S.A.). Generated sequences were then used as queries for the Fusarium-ID v. 1.0^51^ and NCBI^52^ databases to verify the taxonomic identity of the isolates.

In addition, single-locus phylogenetic analyses were conducted using *tef1* gene sequences generated for 30 isolates of the *Fusarium solani* species complex (FSSC) and 17 isolates of the *Fusarium oxysporum* species complex (FOSC). Reference sequences (Supplementary Table S5 and Supplementary Table S6) for these analyses were sourced from previously published phylogenetic studies on the FSSC and the FOSC^16,21,53^ complexes. Representative isolates from the most recent studies on pea and faba bean root rots conducted in the UK, France and Germany^6,11,12^ were also included. The final datasets consisted of 126 *tef1* sequences of the FSSC and 210 of the FOSC (Supplementary Table S5 and Supplementary Table S6). Sequence alignments were generated using MAFFT v.7^54^ and were further adjusted manually with MEGA v6^55^. A Maximum-Likelihood (ML) analysis was conducted with RAxML-VI-HPC v. 7.0.3, employing non-parametric bootstrapping with 1000 replicates via the Cipres portal^56^. For outgroup purposes, *F. udum* (CBS 177.31) and *F. thapsinum* (H05-557 S-1 DCPA) were used (Supplementary Table S5 and Supplementary Table S6). The resulting phylogenetic trees were visualized and edited in FigTree (version 1.4.4; http://tree.bio.ed.ac.uk/software/figtree/) and Adobe Illustrator CS5.1^57^.

### Data analyses

All statistical analyses were conducted in R^58^. Isolation frequencies (% colonized roots) were calculated by dividing the number of roots containing a species by the total number of roots processed. Additionally, if the mean disease severity score within a given field was greater than 3, the field was considered to be seriously affected, a condition that was assessed as the incidence on the field level^6^. The data collected from conventional winter pea fields are presented, however, these data were not included in statistical analyses due to the limited sample size (only 5 fields).

Root rot severity data were analyzed with non-parametric Kruskal-Wallis tests with the pea type (spring vs winter pea), management system (organic vs conventional), sowing pattern (pure vs mixed stands for spring pea only) and year as main factors. Kruskal multiple comparison tests were performed in case of significant effects. Benjamini and Hochberg^59^ stepwise adjustment controlled false discovery rates (FDR) and reduced type I errors. For the isolation frequency data, rare species (< 2% of total root colonization rates) were excluded from the analysis. Generalized linear mixed models with management system, pea type (spring vs winter pea), sowing pattern (pure vs mixed stands for organic spring pea only) and sampling year as factors were employed on proportional data with a binomial response and logit link function^60^. Fields were modeled as random effects, accounting for nested sampling replicates within each field. Model goodness was assessed using Pearson chi-square residual tests, normality checks, and outlier detection (package ‘DHARMa’^61^. Significant main effects were evaluated with ANOVA and Tukey’s correction for post hoc comparisons (*P*< 0.05) (package ‘lsmeans’^62^).

To explore the relationship between the frequencies of the eight most commonly isolated fungal species and yield, root rot incidence on the field level, cropping histories and pedo-climatic factors, Pearson correlation analysis was employed (package ‘Hmisc’^63^. Only statistically significant (p < 0.05) correlation coefficients of ≥ ± 0.30 are reported. In addition, to provide an overview of the soil types for the sampled fields, we employed hierarchical clustering on principal components (HCPC) using the ‘FactoMineR’ package^64^. This approach involves grouping of the fields into clusters based on similarities in soil abiotic properties namely, sand, silt, clay, organic matter content and pH. The R packages ‘maps’^65^, ‘raster’^66^ and ‘ggplot2’^67^ were used to show the coordinates of surveyed fields on a map of Germany. The ggplot2 visualizations were further enhanced with R package ‘ggsn’^68^ which was used to add scale bars and north arrows to the map.

## Electronic supplementary material

Below is the link to the electronic supplementary material.

Supplementary Material 1

## Data Availability

All data are included within the article and its supplementary materials. The complete raw data set generated during this study is available in Supplementary Table S1. Data can also be obtained from the corresponding author upon reasonable request.
